# Supplementary material for: Can Machine Learning Models Detect and Predict Lymph Node Involvement in Prostate Cancer? A Comprehensive Systematic Review
Source: J Clin Med. 2023 Nov 10;12(22):7032. doi: 10.3390/jcm12227032 (PMC10672480; doi:10.3390/jcm12227032)
Supplement: Supplementary file 1 [file jcm-12-07032-s001.zip › jcm-2634480-supplementary.pdf]

Table S1.

Title	Authors	Publication year	Journal	Type	Number of patients	Imaging techniques	AIM	SEGM	AI	RQS	Best results	Conclusions
Preoperative prediction of pelvic lymph nodes metastasis in prostate cancer using an ADC-based radiomics model: comparison with clinical nomograms and PI-RADS assessment.	Liu X. et al. [17]	2022	Abdom Radiol (NY)	Retrospective	474 (between 2017 and 2020). Another cohort of 128 Between 2020 and 2021.	3T mpMRI	develop and test radiomics models based on manually corrected or automatically gained masks on ADC maps for PLNM prediction in Pca patients	2d- 3d U-Net authomatic	AUC and DCA for comparison between the optimal radiomics model and MSKCC, Briganti 2017 nomograms, and PI-RADS assessment	16	The radiomics model based on the mask of automatically segmented prostate obtained the highest AUC among the four radiomics models (0.73 vs. 0.63 vs. 0.70 vs. 0.56). Briganti 2017, MSKCC nomograms, and PI-RADS assessment-yielded AUCs of 0.69, 0.71, and 0.70, respectively, and no significant differences were found compared with the optimal radiomics model (P = 0.605-0.955).	The radiomics model based on the mask of automatically segmented prostate offers a non-invasive method to predict PLNM for patients with PCa. It shows comparable accuracy to the current MKSCC and Briganti nomograms.
Utility of diffusion weighted imaging-based radiomics nomogram to predict pelvic lymph nodes metastasis in prostate cancer.	Liu X. et al. [18]	2022	BMC Med Imaging	Retrospective	84	3T mpMRI	DWI-based radiomics for preoperative PLNM prediction in PCa patients	3d U-Net of LN in DWI	Two preoperative PLNM prediction models with quantitative radiological LN features alone (Model 1) and combining radiological and radiomics features (Model 2) via multiple logistic regression. The visual assessments of junior (Model 3) and senior (Model 4) radiologists were	17	No significant difference between AUCs of Models 1 and 2 (0.89 vs. 0.90; P = 0.573) in the held-out cohort. Model 2 showed the highest AUC (0.83, 95% CI 0.76, 0.89) for PLNM prediction in the LN subgroup with a short diameter ≤ 10 mm compared with Model 1 (0.78, 95% CI 0.70, 0.84), Model 3 (0.66, 95% CI 0.52, 0.77), and Model 4 (0.74, 95% CI	A DWI-based radiomics nomogram incorporating the LN radiomics signature with quantitative radio- logical features is promising for PLNM prediction in PCa patients, particularly for normal-sized LNM

									compared. C-index of the nomogram analysis and DCA were used to evaluate performance and clinical usefulness.		0.66, 0.88). The nomograms of Models 1 and 2 yielded C-index values of 0.804 and 0.910, respectively, in the held-out cohort. The C-index of the nomogram analysis (0.91) and decision curve analysis (DCA) curves confirmed the clinical usefulness and benefit of Model 2.	
Multiparametric MRI-based radiomics model to predict pelvic lymph node invasion for patients with prostate cancer	Zheng H. et al. [19]	2022	Eur Radiol.	Retrospective	244	3T mpMRI	predicting lymph node invasion (LNI), via a radiomics-based machine learning approach.	manual segm of index lesion on T2w seq and ADC map	An integrative radiomics model (IRM) (SVM) performance was measured by AUC, sensitivity, specificity, NPV and PPV.	16	Overall, 17 (10.6%) and 14 (16.7%) patients with LNI were included in training/validation set and testing set, respectively. Shape and first-order radiomics features showed usefulness in building the IRM (integrative radiomics model). The proposed IRM achieved an AUC of 0.915 (95% CI: 0.846–0.984) in the testing set, superior to pre-existing nomograms whose AUCs were from 0.698 to 0.724 ($p < 0.05$).	The proposed IRM could be potentially feasible to predict the risk of having LNI for patients with PCa. With the improved predictability, it could be utilized to assess which patients with PCa could safely avoid ePLND, thus reduce the number of unnecessary ePLND

Development of a Radiomic-Based Model Predicting Lymph Node Involvement in Prostate Cancer Patients	Bourbonne V. et al. [20]	2021	Cancers (Basel)	Retrospective	280	3T e 1,5 T MRI	develop and internally validate a novel LNI risk prediction model based on radiomic features extracted from preoperative multimodal MRI	The index lesion was semi-automatically 3D Slicer.	Radiomic features were extracted from the tumor volumes in ADC map and T2 seq (ComBat harmonization method for inter-site heterogeneity). A prediction model was trained using a neural network approach (Multilayer Perceptron Network) combining clinical, radiomic and all features. AUC and the C-Index for performance	17	The proposed model resulted in a C-Index of 0.89 in the testing set, which was higher than currently available models such as Partin, Roach, Yale, MSKCC, Briganti 2012, and 2017.	The study suggests that radiomic features extracted from preoperative MRI scans combined with clinical features through a neural network could improve LNI risk prediction in PCa.
Radiomics-based prognosis classification for high-risk prostate cancer treated with radiotherapy	Franzese C. et al. [25]	2022	Strahlenther Onkol	Retrospective	157	Non-contrast CT	risk of metastatic progression in HR Pca patients after RT and ADT, considering MFS	Manual of gland only, prostate gland + seminal vescicles, seminal vescicles only.	5 feat clinical + 62 radiomics feat were combined using R platform	13		Radiomic features were able to predict the risk of metastatic progression in high-risk prostate cancer. Combining the radiomic features and clinical characteristics can classify high-risk patients into favorable and unfavorable prognostic groups

A CT-based radiomics model to detect prostate cancer lymph node metastases in PSMA radioguided surgery patients	Peeken JC. et al. [26]	2020	Eur J Nucl Med Mol Imaging	Retrospective	80	Contrast-CT	CT-based radiomic model to predict LNM status using a PSMA radioguided surgery (RGS) cohort with histological confirmation of all suspected lymph nodes (LNs).	LN segmentation was conducted manually using Eclipse 13.0 on the contrast-enhanced diagnostic CT.	156 radiomic features analyzing texture, shape, intensity, and LBP were extracted. Least absolute shrinkage and selection operator (radiomic models) and logistic regression (conventional parameters) were used for modeling	19	A combined radiomic model achieved the best predictive performance with a testing-AUC of 0.95	The best radiomic model outperformed conventional measures for detection of LNM demonstrating an incremental value of radiomic features.
[18F]FMCH PET/CT biomarkers and similarity analysis to refine the definition of oligometastatic prostate cancer	Sollini M. et al. [32]	2021	EJNMMI Res.	Prospective	92	[18F]FMCH PET/CT	evaluate [18F]FMCH PET/CT radiomic analysis in patients with recurrent PCa after primary radical therapy, testing intra-patient lesions similarity in oligometastatic and plurimetastatic PCa, comparing the two most used definitions of oligometastatic disease	Lesions were semi-automatically segmented by the PET VCAR software (GE Healthcare) on a General Electric workstation	supervised and supervised clustering of patients for identifing theprevalence of mets	15		They found a comparable heterogeneity between patients with up to five lesions and plurimetastic patients, while patients with up to three lesions were less heterogeneous than plurimetastatic patients, featuring different cells phenotypes in the two groups. Our results supported the use of a 3-lesion threshold to define oligometastatic PCa.
Radiomic features from PSMA PET for non-invasive intraprostatic tumor discrimination and characterization in patients with	Zamboglou C. [27]	2019	Theranostic	Prospective	20	PSMA PET	tumor discrimination and non-invasive characterization of Gleason score (GS) and pelvic lymph node status.	manually created segmentations of the intraprostatic tumor volume	Coregistered histopathological gross tumor volume. 133 radiomics feat from GTV-Histo of Pca and non-Pca. AUC evaluated the discrimation of Gleason and LN involvement	23	The texture feature QSZHGE was a statistically significant (p<0.01) predictor for PCa patients with GS ≥8 tumors and pN1 status.	Radiomic features derived from PSMA PET discriminated between PCa and non-PCa tissue within the prostate. Additionally, the texture feature QSZHGE discriminated between GS 7 and GS ≥8 tumors and between

intermediate- and high-risk prostate cancer – a comparison study with histology reference												patients with pN1 and pN0 disease.
Machine learning-based analysis of [18F]DCFPyL PET radiomics for risk stratification in primary prostate cancer	Cysouw MCF [28]	2021	Eur J Nucl Med Mol Imaging	Prospective	76	[18F]DCFPyL PET-CT	ability of ML-based analysis of quantitative [18F]DCFPyL PET metrics to predict metastatic disease or high-risk pathological tumor features	primary tumors were delineated using 50–70% peak isocontour thresholds on images with and without partial-volume correction	408 extracted feat analyzed with RF; Gleason, LNI, mets, ECE. Model were trained with PET feat alone and radiomics ones	24	The radiomics-based machine learning models predicted LNI (AUC 0.86 ± 0.15, $p < 0.01$), nodal or distant metastasis (AUC 0.86 ± 0.14, $p < 0.01$), Gleason score (0.81 ± 0.16, $p < 0.01$), and ECE (0.76 ± 0.12, $p < 0.01$). The highest AUCs reached using standard PET metrics were lower than those of radiomics-based models.	Machine learning-based analysis of quantitative [18F]DCFPyL PET metrics can predict LNI and high-risk pathological tumor features in primary PCa patients. These findings indicate that PSMA expression detected on PET is related to both primary tumor histopathology and metastatic tendency.
A machine learning-assisted decision-support model to better identify patients with prostate cancer requiring an extended pelvic lymph node dissection	Hou Y. et al. [23]	2019	BJU Int.	Retrospective	248	3T mpMRI	develop a ML-assisted model for identifying the candidates for ePLND in PCa by integrating clinical, biopsy and precisely defined MRI findings	No radiomic features	18 integrated features using a LR, SVM and RFs models were compared to a MSKCC nomogram. Performance evaluated with AUC and DCA	7	The predictive accuracy of ML-based models, with or without MRI-reported LNI, yielded similar AUCs, while were higher than MSKCC nomogram (0.816; p-value < 0.001).	ML-based model below 15% cutoff, superior to MSKCC nomogram, allows to 50% or more ePLNDs spared at the cost of missing < 3% LNIs.

Integration of clinicopathologic identification and deep transferrable image feature representation improves predictions of lymph node metastasis in prostate cancer	Hou Y et al. [24]	2021	EBioMedicine	Retrospective	401	3TmpMRI	PLNM-Risk calculator to obtain a precisely informed decision about whether to perform extended pelvic lymph node dissection	PCa lesions manually segmented	set of radiologists' interpretations, clinicopathological factors and newly refined imaging indicators from MR images with radiomics machine learning and deep transfer learning algorithms. Its clinical applicability was compared with Briganti and Memorial Sloan Kettering Cancer Center (MSKCC) nomograms.	14	PLNM-Risk achieved good diagnostic discrimination with areas under the receiver operating characteristic curve (AUCs) of 0.93 (95% CI, 0.90-0.96), 0.92 (95% CI, 0.84-0.97) and 0.76 (95% CI, 0.62-0.87) in the training/validation, internal test and external test cohorts, respectively.	PLNM-Risk calculator offers a noninvasive clinical biomarker to predict PLNM for patients with PCa.
Freely Available, Fully Automated AI-Based Analysis of Primary Tumour and Metastases of Prostate Cancer in Whole-Body [18F]-PSMA-1007 PET-CT.	Trägårdh E et al. [29]	2022	Diagnostics (Basel)	Retrospective	660	[18F]PSMA PET-CT	develop and validate a fully automated AI-based method for detection and quantification of suspected prostate tumour/local recurrence, LN, and bone mets from [18F]PSMA-1007 PET-CT	manual	evaluation of total lesion volume and uptake by CNN vs nuclear medicine physician	7	sensitivity of the AI method: 79% for detecting prostate tumour/recurrence, 79% for lymph node metastases, and 62% for bone metastases. On average, nuclear medicine physicians' corresponding sensitivities were 78%, 78%, and 59%, respectively.	The development of an AI-based method for prostate cancer detection with sensitivity on par with nuclear medicine physicians was possible.
Semiautomated pelvic lymph node treatment response evaluation for patients with advanced prostate cancer: based on MET-RADS-P guidelines	Liu X et al. [20]	2023	Cancer Imaging.	Retrospective	162	3T mpMRI	deep learning-based algorithm for semiautomated treatment response assessment of pelvic lymph nodes.	automated segmentation of pelvic lymph nodes.	The performance of the deep learning algorithm was evaluated using the Dice similarity coefficient (DSC) and volumetric similarity (VS). Kappa statistics were used to assess the accuracy and consistency of the	8	The accuracies of automated segmentation-based assessment were 0.92 (95% CI: 0.85–0.96), 0.91 (95% CI: 0.86–0.95) and 75% (95% CI: 0.46–0.92) for target lesions, nontarget lesions and nonpathological lesions, respectively	The deep learning-based semiautomated algorithm showed high accuracy for the treatment response assessment of pelvic lymph nodes and demonstrated comparable performance with radiologists.

									treatment response assessment by the deep learning model and two radiologists			
Freely available artificial intelligence for pelvic lymph node metastases in PSMA PET-CT that performs on par with nuclear medicine physicians	Trägårdh E et al. [30]	2022	Eur J Nucl Med Mol Imaging	Reros pectiv e	211	[18F]PSMA-1007 PET-CT	develop and validate a CNN for detection of PLNM in [18F]PSMA-1007 PET-CT from patients with high-risk PCa.	manual by 3 readers	Suspected pelvic lymph node metastases were marked by three independent readers vs CNN	9	Sensitivity of the AI method for detecting pelvic lymph node metastases was 82%, and the corresponding sensitivity for the human readers was 77% on average.	This study shows that AI can obtain a sensitivity on par with that of physicians with a reasonable number of false positives.
Development and validation of the 3D U-Net algorithm for segmentation of pelvic lymph nodes on diffusion-weighted images	Liu X. et al. [21]	2021	BMC Med Imagin g	Retro specti ve	393	3T mpMRI	evaluate the feasibility of the 3D U-Net algorithm for automated detection and segmentation of LNs on DWI	manual	Segmentation performance was assessed using the Dice score, positive predictive value (PPV), true positive rate (TPR), and volumetric similarity (VS), Hausdorff distance (HD), the Average distance (AVD), and the Mahalanobis distance (MHD) with manual	11	The precision, recall, and F1-score for the detection of suspicious LNs were 0.97, 0.98 and 0.97, respectively. In the temporal validation dataset, the AUC of the model for identifying PCa patients with suspicious LNs was 0.963 (95% CI: 0.892–0.993).	The 3D U-Net algorithm can accurately detect and segment pelvic LNs based on DWI images.

									annotation of pelvic LNs as the reference. The accuracy with which the suspicious metastatic LNs (short diameter > 0.8 cm) were detected was evaluated using the area under the curve (AUC) at the patient level, and the precision, recall, and F1-score were determined at the lesion level.			
Artificial intelligence-based detection of lymph node metastases by PET/CT predicts prostate cancer-specific survival.	Borrelli P. et al. [31]	2021	Clin Physiol Funct Imaging	Retrospective	399	PET/CT	developing and validating an AI-based tool for detection of lymph node lesions on PET/CT	authomatic by Organ CNN	The tool consisted of CNN using complete PET/CT scans as inputs. In the test set, the AI-based LN detections were compared to those of two independent readers. The association with PCa-specific survival was investigated.	7	The AI-based tool detected more lymph node lesions than Reader B (98 vs 87/117; p=0.045) using Reader A as reference. AI-based tool and Reader A showed similar performance (90 vs 87/111; p=0.63) using Reader B as reference.	This study shows the feasibility of using an AI-based tool for automated and objective interpretation of PET/CT images that can provide assessments of lymph node lesions comparable with that of experienced readers, and prognostic information in PCa patients.
